# Nicotine levels in emissions from heat sticks without tobacco exceed health-based advisory values: A chemical-analytical study

**DOI:** 10.18332/tid/219209

**Published:** 2026-07-26

**Authors:** Charlotte G.G.M. Pauwels, Caroline Versluis, Reinskje Talhout, Anne Havermans

**Affiliations:** 1 Department of Chemical Analysis, Tobacco and Drugs, Centre for Health Protection, National Institute for Public Health and the Environment, Bilthoven, The Netherlands

**Keywords:** nicotine heat stick, HTP, nicotine, advisory value

## Abstract

**INTRODUCTION:**

Heated tobacco products (HTP) are tobacco-containing sticks that have been on the market for some time and are heated in a device to release a nicotine-containing aerosol for inhalation. Recently, new sticks have emerged that contain nicotine but no tobacco and are available in several flavors. These nicotine sticks closely resemble tobacco sticks in appearance and use, but contain nicotine, a non-tobacco carrier such as cellulose or Rooibos tea leaves, and other additives, including flavorings. As they do not contain tobacco, these products are currently not regulated under the European Union Tobacco Products Directive (EU TPD). Since the nicotine levels in these products are unknown, this study aimed to measure the nicotine concentrations in both the contents and emissions of nicotine heat stick brands marketed in the Netherlands.

**METHODS:**

This chemical-analytical study was conducted in March 2025. Two flavor variants from two major brands were analyzed for nicotine content in both sticks and emissions. Emissions were generated and collected with a smoking machine following the World Health Organization (WHO) Intense regime. All extracts were analyzed for nicotine by gas chromatography-mass spectrometry.

**RESULTS:**

All sticks contained nicotine (3.2–3.8 mg per stick), with no detectable 6-methylnicotine. Emissions analysis showed each stick released 0.7–1.0 mg nicotine under standardized conditions.

**CONCLUSIONS:**

Nicotine heat sticks without tobacco deliver nicotine doses comparable to HTPs. The measured levels exceed health-based advisory values for nicotine exposure from nicotine products without tobacco for inhalation, as reported by the Dutch National Institute for Public Health and the Environment (RIVM), by a factor of 18–25. If these advisory values are implemented in tobacco legislation, nicotine heat sticks products will no longer be permitted. These limits would apply to all nicotine products without tobacco for inhalation that are not covered by the EU TPD, except e-cigarettes.

## INTRODUCTION

Nicotine heat sticks without tobacco have recently become available in many countries^1^. These products are designed to be used in the same electronic heating devices as tobacco sticks – the so-called heated tobacco products (HTPs)^2^. While HTPs contain processed tobacco as filler, nicotine heat sticks typically contain nicotine blended with flavorings, humectants, and carrier materials such as cellulose or tea leaves, according to the manufacturer^1,3,4^. Upon heating, the nicotine heat sticks release aerosolized nicotine and flavor compounds, which are inhaled by the user. Moreover, nicotine heat sticks are visually and functionally identical to HTPs; the only distinguishing feature is the absence of tobacco. The market for these products appears to be growing. This growth can be attributed to the fact that they are marketed as less harmful than traditional tobacco products, to the wide range of flavors – such as fruit and menthol – offered by various manufacturers in attractive, modern packaging, and to the perception that these products can be used more discreetly, since they produce less emissions or odor than when smoking cigarettes^3,4^.

Despite the absence of tobacco, these products are not harmless and contain, among others, nicotine, glycerol, and flavorings^4^. Nicotine is a well-established addictive substance with known adverse effects on cardiovascular, respiratory, and neurological health^5-7^. The expansion of this product category, combined with marketing that emphasizes appealing flavors and sleek packaging while implying reduced risk through terms such as ‘tobacco-free’, raises concerns about increased uptake – particularly among adolescents, young adults, and non-smokers.

A significant regulatory gap currently exists in Europe for nicotine-containing products without tobacco. E-cigarettes are the only products without tobacco regulated within the European Union Tobacco Products Directive (EU TPD), which allows a maximum nicotine content of 20 mg/mL for e-cigarettes^8^. In addition, the EU TPD prohibits characterizing flavors for cigarettes, roll-your-own (RYO) cigarettes, and HTPs, but these restrictions do not apply to nicotine heat sticks without tobacco^8^.

The Netherlands is seeking to address this issue by proposing maximum limits for nicotine emissions from nicotine products without tobacco for inhalation, such as nicotine heat sticks, based on health risk analysis^9^. This limitation can be implemented at the national level, as these products are not regulated by the EU TPD^10^, and will apply to all inhaled nicotine products except e-cigarettes, which are regulated separately in the EU TPD. A recent publication of the Dutch National Institute for Public Health and the Environment (RIVM) reports health-based advisory values for exposure to nicotine from nicotine products without tobacco for inhalation^7^: the lowest levels, whereby no negative health effects of nicotine are expected, are 0.028 mg nicotine emitted per product (systemic toxicity) and 0.07 mg/L in aerosol (local toxicity). There is currently a lack of data regarding the nicotine content and emission of nicotine heat sticks, making it unclear whether these products would exceed health-based maximum limits. Additionally, it remains unclear whether these heat sticks possibly contain structural analogues of nicotine, such as 6-methylnicotine (6-MN). 6-MN has previously been used as a nicotine substitute in oral products and may potentially be used in other products in the future as a means to circumvent nicotine legislation^11^. The present study was conducted to measure nicotine levels in both the contents and emissions of nicotine heat-stick brands marketed in the Netherlands. To ensure the methods are future-proof, 6-MN was included in the analyses. The measurements of nicotine and 6-MN were compared to newly derived advisory values intended to protect users from adverse health effects from inhaled nicotine^7^. By providing these data, this study aims to inform evidence-based policy and support the development of effective regulations for nicotine-containing products that currently lie outside the scope of the EU TPD.

## METHODS

This chemical-analytical study was conducted in March 2025.

### Nicotine stick selection

**Figure 1:**
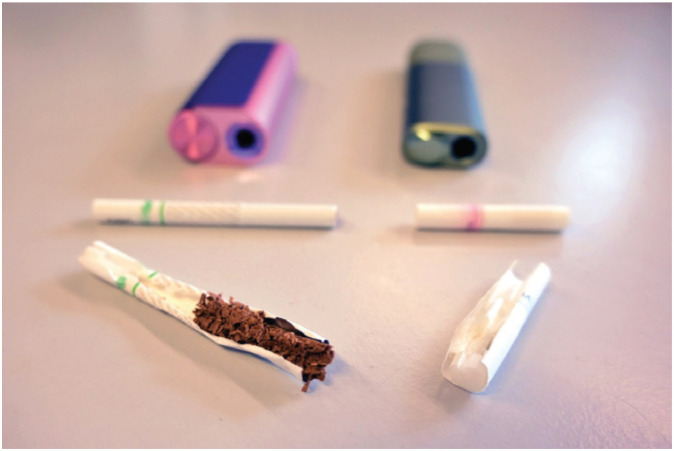
Two different nicotine sticks cut open; left a Veo stick, right a Levia stick

Several tobacco manufacturers currently produce nicotine heat sticks for use in heated tobacco devices^3,4,12^. For this laboratory study, products were selected from two major manufacturers with known availability on the Dutch market: Philip Morris International (PMI) and British American Tobacco (BAT) (Figure 1).

PMI produces Levia nicotine heat sticks, which are designed for use with the IQOS ILUMA heating device and resemble their tobacco-containing Heets and Terea sticks in appearance. According to the manufacturer, Levia sticks do not contain tobacco but are composed of cellulose (plant fiber), nicotine, and propylene glycol^4^. Each stick consists of a mouthpiece with a filter, and a paper tube filled with a white cellulose sheet wrapped around a metal plate intended for induction heating^13^. Two variants were analyzed: Levia Electro Rouge (blueberry and floral flavor) and Levia Island Beat (creamy mint flavor). The nicotine content is not specified on the product packaging.

BAT produces Veo nicotine sticks for use with the Glo heating device that uses conduction heating (heating the stick through direct contact with a heating element). These sticks are comparable in appearance to their Neo tobacco sticks. Veo sticks are composed of a mouthpiece with a filter containing an integrated flavor capsule, and a paper tube filled with processed Rooibos tea leaves, packaged in aluminium foil. For this study, the two variants with flavors similar to those of the PMI sticks were selected: Veo Green Click and Veo Purple Click^3^. Veo Green Click is described as having a sweet, creamy peppermint flavor, while Veo Purple Click has a blueberry and creamy aroma. Like the Levia sticks, the nicotine content of Veo sticks is not specified on the packaging.

### Nicotine and 6-MN analysis in nicotine heat stick filler

To determine the nicotine content in the nicotine heat sticks, each stick was cut open longitudinally, and the portion intended for heating by the device (i.e. the rod) was separated from the mouthpiece. For Veo sticks, the flavor capsule in the mouthpiece was crushed before extraction.

The rod and the mouthpiece of one stick were extracted separately using 10 mL of a solution containing 70% methanol (Biosolve, NL) and 30% acetonitrile (Sigma, DE). Xylene-D10 and heptadecane (Sigma, DE) were added as internal standards, following the protocol described in WHO TobLabNet SOP 15^14^, which is used for the determination of nicotine, glycerol, and propylene glycol in HTPs. The separate extraction solutions were shaken for 60 minutes at 120 rpm, after which an aliquot was transferred to a vial for analysis. Extractions were performed in triplicate for each product variant.

The extracts were analyzed using a non-targeted screening by gas chromatography-mass spectrometry (GC-MS) as described in WHO TobLabNet SOP 16^15^. In short, the analysis was performed on an Agilent 7890 GC/5977C MSD equipped with a J&W inertcap aquatic-2 column (60 m × 250 µm × 1.4 µm) under the following conditions: injection volume of 1 µL, split injection mode with a ratio of 10:1, and helium as carrier gas. The total runtime was 41 minutes, with an initial oven temperature of 50°C, ramping at 10°C per minute to a final temperature of 250°C. The mass range was set from 30 m/z to 250 m/z. Nicotine (162.23 g/mol, Acros, BE) and 6-MN (176.26 g/mol, LGC, UK) were identified and quantified, if present.

### Nicotine emission measurement from nicotine heat sticks

To collect emissions from the nicotine sticks, the products were heated in their corresponding devices (IQOS ILUMA One (model M0004) and Glo Hyper Pro (model no. G6100)) using a 4-port linear puffing machine (Borgwaldt, Körber Technologies GmbH, Hamburg, Germany). The flavor capsule in the mouthpiece of the Veo sticks was crushed directly before machine smoking. Since the puffing machine could not automatically activate the heating devices, the devices were manually switched on before each smoking session. The Glo heating device standard mode heats up to 240–250°C, and the ILUMA device heats up to 300–350°C according to the manufacturers^16,17^.

In the absence of a validated protocol for smoking nicotine sticks, the standardized WHO Intense smoking regime was applied^18,19^. According to this protocol, the smoking machine delivers one puff every 30 seconds, each lasting 2 seconds, with a puff volume of 55 mL. Ten puffs were taken per stick, resulting in a total emission volume of 550 mL per stick (10 × 55 mL). The Glo device automatically shuts off after four minutes, making it impossible to take ten puffs at 30-second intervals. To reach a total emission volume of 550 mL per stick, the inter-puff interval for Glo was adjusted to 20 seconds, allowing for ten puffs within four minutes and 18 seconds.

Emissions from two sticks were collected on a 44 mm diameter glass fiber Koerber filter. For each stick variant, this process was repeated three times. After collection, the filters were extracted according to ISO 10315^20^. The collected extracts were then analyzed for nicotine and 6-MN using the screening via GC-MS, as described above.

## Results

Nicotine was detected in all heat sticks, whereas 6-MN was not detected in any sample (Table 1). Specifically, Levia sticks (PMI) contained an average of 3.8 mg nicotine per stick, while Veo sticks (BAT) contained an average of 3.3 mg nicotine per stick (Table 1). Within each brand, the two flavor variants contained similar amounts of nicotine per stick.

**Table 1:** Nicotine content in sticks and nicotine emission from four nicotine heat stick variants of two brands

*Stick variant*	*UFI code*	*Heating* *device*	*Nicotine in* *mouthpiece* *(mg)*	*Nicotine in* *rod* *(mg)*	*Total nicotine* (mg/stick)*	*Nicotine in* *emission (mg/* *stick)*	*6-MN*
**Levia Island Beat**	MT2Q-27SV-2EQ8-VP76	Iluma	0.13 ± 0.01	3.59 ± 0.15	3.72 ± 0.14	0.70 ± 0.09	ND
**Levia Electro Rouge**	VQH8-Q8KT-PEQ1-7CDN	Iluma	0.12 ± 0.01	3.68 ± 0.15	3.79 ± 0.14	0.67 ± 0.06	ND
**Veo Green Click**	N5JM-F68V-VN54-CQDJ	glo	0.09 ± 0.01	3.37 ± 0.30	3.46 ± 0.31	0.98 ± 0.03	ND
**Veo Purple Click**	6JAY-7C89-GQK4-VQNA	glo	0.08 ± 0.00	3.15 ± 0.08	3.22 ± 0.08	0.95 ± 0.04	ND

Data are presented as mean ± standard deviation (SD). The Unique Formula Identifier (UFI) codes of the products included in the experiment are 16-character alphanumeric codes printed on product labels that uniquely identify the specific chemical mixture in the product for regulatory purposes. 6-MN: 6-methylnicotine. ND: not detected.

* Total nicotine per stick is the sum of the nicotine in the mouthpiece and the nicotine in the rod, calculated from the raw data.

Analysis of nicotine emissions from machine smoking showed that Levia sticks emitted an average of 0.7 mg of nicotine per stick, while Veo sticks emitted an average of 1.0 mg of nicotine per stick.

## DISCUSSION

The present study was conducted to measure the levels of nicotine in both the contents and emissions of nicotine heat stick brands marketed in the Netherlands. The results of this study demonstrate that both Levia sticks (PMI) and Veo sticks (BAT) contain nicotine, but no detectable amounts of 6-MN. Levia (PMI) and Veo (BAT) sticks contained 3.8 mg and 3.3 mg nicotine per stick, respectively. For comparison, manufacturer data (submitted to the European Common Entry Gate (EU-CEG) system^21^ for products active in the Netherlands as of 1 July 2025, show that conventional cigarettes contain 15.9–18.8 mg nicotine per gram of unburned tobacco filler^8^. With an average cigarette containing about 0.6 g of tobacco, this equals roughly 9.5–11.3 mg nicotine per cigarette^8^.

Despite differences in stick design, heating device, and operating temperature between brands, the results of this study show that users of both brands may be exposed to comparable levels of nicotine emission: Veo sticks emitted 1.0 mg, and Levia sticks 0.7 mg nicotine per stick on average. Machine smoking measurements of HTPs (Yellow HEET) have found similar results, e.g. 1.2 ± 0.14 mg/stick, while cigarette smoke generally contains (slightly) higher levels, e.g. (on average) 1.75 mg/cigarette, when measured using the WHO Intense method^18,22^.

These findings indicate that nicotine heat sticks without tobacco can deliver substantial amounts of nicotine, with emissions in the same range as tobacco-containing heat sticks and somewhat lower than conventional cigarettes. This suggests that users of these new nicotine sticks are exposed to significant doses of nicotine, potentially leading to similar risks of addiction and health effects as with traditional tobacco products.

With regard to advisory values for the maximum emission of nicotine and 6-MN from nicotine products without tobacco for inhalation, Levia and Veo sticks, as nicotine products without tobacco for inhalation, can be directly compared to the health-based advisory values in the recent publication of the RIVM^7^. The lowest measured emission (Levia) was 0.7 mg nicotine per stick, 25 times higher than the systemic advisory value. The maximum nicotine concentration in the emission was 1.27 mg/L, 18 times higher than the advisory value for local toxicity effects. These findings show that both Levia and Veo sticks strongly exceed the thresholds recommended by RIVM.

### Strengths and limitations

This study is the first to present nicotine measurements in the stick and in the emissions of nicotine heat sticks. Analyses were conducted using a WHO protocol, and results were compared to advisory levels. However, some limitations should be acknowledged. The analysis was restricted to four stick variants from two brands available on the Dutch market, and focused only on nicotine and 6-MN. These products and compounds may not represent the full range of products, formulations, or other relevant compounds available from all manufacturers in the Netherlands and internationally. Additionally, batch-to-batch variability, product aging, and changes in emissions over repeated use were not taken into account. Further, in the absence of a validated protocol for smoking nicotine sticks, the standardized WHO Intense smoking regime was applied. However, because the Glo device could not accommodate this protocol, an adapted regime was used to achieve a similar total puff volume, and this may have introduced differences between the brands. It should also be noted that, although machine protocols were used in this study, machine-measured emissions do not directly translate to real-world user exposure, which can vary substantially depending on user behavior. Notably, recent research by Davigo et al.^18^ suggests that even more intense smoking regimes than the WHO Intense method may be warranted for laboratory assessment of heat stick emissions^18,23,24^.

### Implications

In terms of regulatory implications, recently, the Dutch Senate (Eerste Kamer) adopted a legislative amendment bringing nicotine products without tobacco other than e-liquids under the scope of the Tobacco and Smoking Products Act (TSPA)^10^. A ministerial regulation can establish specific product requirements, such as limits on nicotine content or emissions. When the advisory values from RIVM report 2025-0067 are incorporated into this regulation, the nicotine heat sticks analyzed in this study would not comply with the proposed requirements. Consequently, when these thresholds are implemented, this type of product will be subject to regulatory actions, which could lead to reformulation or removal from the Dutch market. While Dutch regulations exclude e-cigarettes, which are regulated separately in the EU TPD, other jurisdictions may choose to include all nicotine-containing products without tobacco, in order to limit exposure to nicotine from such products.

## CONCLUSIONS

This study demonstrates that nicotine heat sticks designed for use in heating devices deliver nicotine doses similar to those of HTPs and traditional cigarettes. These products produce emissions that significantly exceed recently established health-based advisory values for both systemic and local effects from nicotine exposure.

## Data Availability

The data supporting this research are available from the authors on reasonable request.
